# Identification of the Risk Genes Associated With Vulnerability to Addiction: Major Findings From Transgenic Animals

**DOI:** 10.3389/fnins.2021.811192

**Published:** 2022-01-12

**Authors:** Chloe J. Jordan, Zheng-Xiong Xi

**Affiliations:** ^1^Division of Alcohol, Drugs and Addiction, Department of Psychiatry, McLean Hospital, Harvard Medical School, Belmont, MA, United States; ^2^Molecular Targets and Medications Discovery Branch, National Institute on Drug Abuse, Intramural Research Program, Baltimore, MD, United States

**Keywords:** alcohol, cocaine, opioids, risk genes, dopamine, D_2_ receptor, D_3_ receptor, mGluR_2_

## Abstract

Understanding risk factors for substance use disorders (SUD) can facilitate medication development for SUD treatment. While a rich literature exists discussing environmental factors that influence SUD, fewer articles have focused on genetic factors that convey vulnerability to drug use. Methods to identify SUD risk genes include Genome-Wide Association Studies (GWAS) and transgenic approaches. GWAS have identified hundreds of gene variants or single nucleotide polymorphisms (SNPs). However, few genes identified by GWAS have been verified by clinical or preclinical studies. In contrast, significant progress has been made in transgenic approaches to identify risk genes for SUD. In this article, we review recent progress in identifying candidate genes contributing to drug use and addiction using transgenic approaches. A central hypothesis is if a particular gene variant (e.g., resulting in reduction or deletion of a protein) is associated with increases in drug self-administration or relapse to drug seeking, this gene variant may be considered a risk factor for drug use and addiction. Accordingly, we identified several candidate genes such as those that encode dopamine D_2_ and D_3_ receptors, mGluR_2_, M_4_ muscarinic acetylcholine receptors, and α_5_ nicotinic acetylcholine receptors, which appear to meet the risk-gene criteria when their expression is decreased. Here, we describe the role of these receptors in drug reward and addiction, and then summarize major findings from the gene-knockout mice or rats in animal models of addiction. Lastly, we briefly discuss future research directions in identifying addiction-related risk genes and in risk gene-based medication development for the treatment of addiction.

## Introduction

While many individuals are exposed to alcohol and other substances, not all develop substance use disorders (SUD; Marel et al., 2019). Multiple factors have been associated with alcohol use disorder (AUD) and SUD, including individual traits such as impulsivity, stress reactivity, novelty seeking, family history, socioeconomic status, and trauma exposure (Dawe and Loxton, 2004; Nower et al., 2004; Tucci et al., 2010; Marel et al., 2019; Cook et al., 2021). In addition, alcohol and SUD are associated with variations in specific gene expression (Goldman et al., 2005). Understanding such risk genes can prompt the development of new pharmacotherapies for the prevention and treatment of AUD and SUD.

Early family-based linkage studies indicate that people with first-degree relatives with AUD or SUD are generally at greater risk for the development of substance use and addiction (Goldman et al., 2005). However, these studies cannot distinguish whether the difference in addictive behavior among family members is caused by genetic or environmental factors. This limitation has been addressed by twin and adoption studies based upon the assumption that similarity between offspring and biological parents is suggestive of genetic influences on that behavior, while similarity between offspring and adoptive parents is suggestive of environmental influences (Cadoret et al., 1986; Agrawal and Lynskey, 2008). Reviews of the evidence from twin and adoption studies indicate that the heritability of addictive disorders range from a low of 0.39 (hallucinogens) to a high of 0.72 (cocaine), with drugs like alcohol, opioids, and smoking in between (Goldman et al., 2005; Schuckit, 2009). However, such family-based studies cannot identify specific genes underlying vulnerability to AUD or substance use disorders.

While genetic linkage and association approaches such as Genome-Wide Association Studies (GWAS) have been widely used to identify candidate genes in humans (Hall et al., 2013, 2020; Serafine et al., 2021), to date few genes identified by GWAS have been verified by clinical or preclinical studies. Another major approach used to identify specific risk genes has been the examination of candidate genes that encode proteins that are critically involved in the pharmacological action of misused drugs, such as genes related to brain dopaminergic, GABAergic, opioid, and cholinergic systems (Hall et al., 2013; Serafine et al., 2021). Transgenic approaches have been valuable in identifying genes and proteins relevant to SUD, such as μ opioid receptors (MOR) for opioid action, dopamine transporters (DAT) for cocaine action, and the vesicular monoamine transporter 2 (VMAT2) for amphetamine and methamphetamine action (Sora et al., 2010). However, findings in transgenic animals do not necessarily indicate that variation in these genes constitute addiction risk factors; instead, some genes/proteins may simply act as the targets of addictive drugs, or even as protective factors against SUD. For example, genetic deletion of DAT, dopamine D_1_ receptors or mGluR_5_ caused a dramatic decrease in cocaine self-administration (Rocha et al., 1998; Chiamulera et al., 2001; Caine et al., 2007; Thomsen et al., 2009a,b), suggesting that a reduction in these genes is unlikely to lead to risk for SUD. Conversely, a few other mouse strains, such as dopamine D_2_ or D_3_ receptor knockouts (KO) express elevated drug-taking and drug-seeking behaviors (Caine et al., 2002; Song et al., 2012), suggesting that a reduction in such gene expression may increase vulnerability to drugs of use, promoting the development of SUD.

In this mini-review, we focus on a few candidate genes that may be particularly associated with the development of drug use and addiction. A guiding hypothesis is that if a particular gene variant (e.g., resulting in a reduction or absence of a protein) is associated with an increase in drug taking and drug seeking in self-administration paradigms, this gene variant may constitute a risk factor for drug use and addiction. Based on this hypothesis, we identified several genes such as dopamine D_2_ or D_3_ receptors, mGluR_2_, M_4_ muscarinic acetylcholine receptors, and α_5_ nicotinic acetylcholine receptors, that may act as potential SUD risk genes. Here, we briefly describe the functional role of these genes in drug reward and addiction and then summarize major findings from these transgenic gene KO animals. Lastly, we briefly discuss future research directions in further identifying the addiction risk genes and the importance of gene-based medication development for the treatment of addiction.

## Reduced D_2_ and D_3_ Receptor Expression Are Risk Factors for Stimulant, Opioid and Alcohol Use

There are five known dopamine receptor subtypes, categorized into excitatory ‘D_1_-like’ (D_1_ and D_5)_ receptors and inhibitory D_2_-like (D_2_, D_3_, and D_4_) receptors, based on their intracellular signaling effects (Albert et al., 1990; Bouthenet et al., 1991; Huang and Kandel, 1995; Robinson and Caron, 1997; Rankin et al., 2006; Beaulieu and Gainetdinov, 2011). Of these, little is known regarding the role of the D_5_ receptors in SUD, while deletion of D_4_R appears to have little impact on SUD risk and deletion of the D_1_R may represent a protective, rather than risk factors for drug use. Accordingly, we briefly describe findings in transgenic animals with manipulation of D_1_, D_4_, and D_5_ receptors in relation to addiction-related behaviors, but focus primarily on the inhibitory D_2_ and D_3_ receptors, which appear to match the risk gene profile for drug misuse and addiction.

### D_1_ Receptors

The D_1_R is expressed widely in the brain, including striatum, nucleus accumbens, substantia nigra, olfactory bulb, amygdala, prefrontal cortex, hippocampus, cerebellum, thalamus and hypothalamus (Aizman et al., 2000; Beaulieu and Gainetdinov, 2011). Extensive investigations of the role of D_1_R in substance use have been conducted (Hummel and Unterwald, 2002). The D_1_R is critically important in mediating cocaine’s action, as blockade of D_1_R reduces sensitization to cocaine, suppresses cocaine conditioned place preferences and cocaine seeking during self-administration, and decreases cocaine-related changes in striatal gene expression (McCreary and Marsden, 1993; Caine et al., 2007; Guan et al., 2009; Kim and Lattal, 2019; Gu et al., 2020).

Human *in situ* hybridization studies did not show significant changes in D_1_ expression in post-mortem brains with a history of cocaine use (Meador-Woodruff et al., 1993). Mice lacking D_1_ receptors die shortly after weaning unless they are provided hydrated food (Drago et al., 1994). At basal levels, D_1_R KO mice show higher dopamine turnover and higher levels of dopamine in the midbrain compared to controls (El-Ghundi et al., 1998). Mice with deletion of the D_1_R fail to acquire cocaine self-administration, while opioid and food self-administration are comparably unaffected (Caine et al., 2007). Mutation or KO of D_1_R also reduces cocaine-induced locomotor activity and nucleus accumbens dopamine, and suppresses cocaine-induced changes in gene expression in the nucleus accumbens and caudate/putamen (Xu et al., 1994; Zhang et al., 2002; Karlsson et al., 2008). However, other studies suggest that D_1_R KO mice do not show significant differences from wild type mice in cocaine place preferences (Miner et al., 1995). In addition, D_1_R mutant mice showed reduced sensitivity to morphine-induced locomotor activity and did not acquire morphine conditioned place preferences compared to both wild type and D_3_R KO mice (Becker et al., 2001; Wang et al., 2015). Similarly, D_1_R KO mice do not show methamphetamine-related hyperthermia or neurotoxicity (Ares-Santos et al., 2012). D_1_R KO mice also fail to show amphetamine-induced locomotor sensitization (El-Ghundi et al., 1998). With respect to alcohol, D_1_R KO mice exhibit reduced alcohol consumption and place preferences compared to wild type controls in free-choice limited and continuous access paradigms (El-Ghundi et al., 1998). Taken together, these findings from D_1_R-KO mice suggest that the D_1_R is fundamental to cocaine seeking behaviors, but may also play a more subtle role in opioid, food, and other reward-seeking. Reduced D_1_R expression appears to be protective against drug reward-related behaviors.

### D_2_ Receptors

The role of D_2_R in addictions has also been studied and reviewed extensively (Volkow and Morales, 2015). Briefly, in Positron Emission Tomography (PET) imaging studies, D_2_R levels predict “liking” the effects of psychostimulants such as methylphenidate (Volkow et al., 1999). Chronic cocaine use/misuse as well as alcohol misuse attenuates D_2_R/D_3_R availability in the striatum and blunts dopamine responses to psychostimulant administration (Volkow et al., 2014, 2017). In post-mortem studies, people with alcohol use disorder showed reduced D_2_R binding in the cortex (Tupala et al., 2004). Alcohol preferring rodents also show lower D_2_R mRNA and protein expression in the nucleus accumbens, striatum, and hippocampus (Thanos et al., 2004; Bice et al., 2008). Administration of D_2_R antagonists to rats causes a compensatory increase in cocaine self-administration (Caine et al., 2002).

The impact of D_2_R deletion on drug reward-related behaviors is mixed. In electrical brain stimulation reward paradigms, stimulation of the forebrain bundle in the lateral hypothalamus is defined as rewarding, since rodents will lever-press to earn this stimulation [also known as operant intracranial self-stimulation (ICSS)]. ICSS is reduced by deletion of D_2_R in D_2_R KO mice, indicating that D_2_R KO mice require more stimulation to sustain reward-related responding (Elmer et al., 2005). Strikingly, D_2_R KO mice self-administer more cocaine than wild type controls (Caine et al., 2002) and exhibit stronger cocaine place preferences (Bello et al., 2011), suggesting that reduced D_2_R expression in the brain could be a risk factor in promoting cocaine use. By contrast, D_2_R KO mice exhibit attenuated locomotor sensitization to cocaine and methamphetamine (Solis et al., 2021), reduced acute stimulant effects of methamphetamine compared to controls (Kelly et al., 2008), and reduced responding for food (Caine et al., 2002; but see Bello et al., 2011), suggesting an important role of D_2_R in mediating psychostimulant and non-drug reward.

D_2_R KO mice do not respond differently for intravenous (i.v.) saline vs. i.v. morphine, and fail to increase responding for i.v. morphine under a progressive ratio schedule (Elmer et al., 2002). However, both cocaine and morphine increased dopamine levels in the striatum to a greater degree in D_2_R KO mice compared to wild type controls (Rouge-Pont et al., 2002; Bello et al., 2011). With respect to alcohol, D_2_R KO mice consume and respond less for alcohol compared to wild type controls, and show no evidence of place preferences for alcohol (Phillips et al., 1998; Cunningham et al., 2000; Risinger et al., 2000). Together, these findings suggest the D_2_R plays an important role in regulating behavioral responses to addictive drugs. As such, reduced D_2_R expression in humans could be a risk factor for the development of drug misuse.

### D_3_ Receptors

Although D_1_ and D_2_ receptors have received the most attention for their involvement in SUD (Volkow and Morales, 2015), the D_3_R subtype has also received increasing attention for its role in drug use and addiction, and as a therapeutic target for the treatment of SUD (Heidbreder and Newman, 2010; Sokoloff and Le Foll, 2017; Galaj et al., 2020). In contrast to the lack of changes in D_1_ and D_2_ receptor expression in post-mortem human brains (Meador-Woodruff et al., 1993), cocaine overdose victims displayed significant D_3_R overexpression in the striatum, particularly the ventral nucleus accumbens (Staley and Mash, 1996). In addition, people who used cocaine and methamphetamine also exhibited increased D_3_R availability and binding in the midbrain (Matuskey et al., 2014; Boileau et al., 2016; Prieto, 2017), suggesting that D_3_R may play an important role in psychostimulant misuse.

The D_3_R is an inhibitory G-protein coupled receptor expressed in midbrain dopamine neurons, striatal GABAergic neurons, and glutamate neurons in amygdala, hippocampus, and prefrontal cortex (Diaz et al., 2000; Li and Kuzhikandathil, 2012; Clarkson et al., 2017). D_3_R regulates cocaine effects on extracellular signal-regulated kinase (ERK) and inhibits NMDA activation of CaMKIIα (Jiao et al., 2007). Chronic ethanol intake increases D_3_R expression in the striatum (Leggio et al., 2014). Lentiviral overexpression of D_3_R in the nucleus accumbens suppresses cocaine-induced locomotor sensitization (Bahi et al., 2005), augments voluntary ethanol intake, and enhances alcohol place preferences (Bahi and Dreyer, 2014). By contrast, lentiviral knockdown of D_3_R in the nucleus accumbens reduces voluntary alcohol consumption and blocks alcohol place preferences in rats (Bahi and Dreyer, 2014).

D_3_R-deficient mice show higher basal dopamine levels in the striatum (Koeltzow et al., 1998), increased DAT expression, and higher basal locomotor activity and rearing, most notably in response to novel environments (Accili et al., 1996; Boyce-Rustay and Risinger, 2003; Li et al., 2010). D_3_R KO mice are also more impulsive on the 5-choice serial reaction time task (5-CSRTT) (Wang et al., 2017). These findings suggest that presynaptic D_3_R tonically modulates dopamine release. Deletion of presynaptic D_3_R may cause an increase in basal dopamine release and altered dopamine responses to various stimuli (Song et al., 2012).

While some evidence suggests that the D_3_R may play a subtle or very small role in cocaine self-administration (Caine et al., 2012), other findings suggest that genetic deletion of D_3_R in mice causes a significant increase in cocaine self-administration behaviors, coupled with reductions in cocaine-induced dopamine release in the nucleus accumbens compared to controls (Song et al., 2012). Specifically, mice with D_3_R deletion exhibit increases in cocaine intake during acquisition and maintenance of self-administration, upward shifts in cocaine dose-response curves, increased motivation to earn cocaine under a progressive ratio schedule of reinforcement, and faster extinction of cocaine-seeking behaviors (Song et al., 2012). Cocaine cue-conditioned hyperactivity is also enhanced following D_3_R KO (Le Foll et al., 2002). Furthermore, D_3_R KO mice show faster acquisition of cocaine and amphetamine place preferences (Xu et al., 1997; Kong et al., 2011; but see Song et al., 2013), along with delayed extinction of place preferences (Chen and Xu, 2010). These altered behavioral responses to cocaine coincided with elevated ERK activation in the nucleus accumbens and prefrontal cortex (Chen and Xu, 2010), and increased c-fos responses to cocaine in the striatum (Carta et al., 2000). In addition, D_3_R mutant mice (Xu et al., 1997) express increased activation of ERK and CAMKIIα in the nucleus accumbens, amygdala, and prefrontal cortex in response to low doses of cocaine (Kong et al., 2011), as well as increased locomotor responses to low, but not high, doses of cocaine (Xu et al., 1997) compared to wild type controls. D_3_R-deleted mice also demonstrate increased cocaine-induced stereotypic behavior, elevated locomotor sensitization to cocaine and methamphetamine, and elevated c-fos and dynorphin in the nucleus accumbens and striatum (Carta et al., 2000; Chen et al., 2018), suggesting that decreased D_3_R expression may constitute a risk factor in the development of cocaine use and addiction.

The indication that low D_3_R expression may constitute a SUD risk factor is further supported by the findings that D_3_R deletion increases heroin seeking and heroin intake. Specifically, mice with KO of the D_3_R took more heroin, exhibited upward-shifted heroin dose-response functions, and increased motivation to self-administer heroin under a progressive ratio schedule compared to controls, as well as elevated heroin seeking during extinction and reinstatement testing (Zhan et al., 2018). D_3_R-deleted mice show stronger place preferences for morphine, even at doses that do not induce CPP in wild type mice (Narita et al., 2003; Frances et al., 2004). However, D_3_R KO mice express less locomotor sensitization following acute and repeated morphine exposure (Li et al., 2010; Lv et al., 2019). D_3_R KO mice do display increased basal extracellular dopamine levels in the nucleus accumbens and reduced dopamine responses to heroin (Song et al., 2012; Zhan et al., 2018). Furthermore, D_3_R KO mice have longer latencies to tail-flick in response to a painful stimulus, potentiated morphine-induced analgesia, lower morphine tolerance and reduced naloxone-precipitated withdrawal signs compared to wild type mice (Li et al., 2012). These results suggest that D_3_R is involved not only in the rewarding effects of opioids but also opioid analgesia, tolerance, and withdrawal. As such, low D_3_R expression may be a risk factor for opioid misuse as well as stimulant misuse.

However, not all evidence supports the above conclusions. Stimulant-related locomotor activity findings are mixed in D_3_R KO mice. Some studies report that D_3_R KO mice develop normal amphetamine-related sensitization (Harrison and Nobrega, 2009). By contrast, other studies indicate D_3_R deletion attenuates methamphetamine-related locomotor activity and sensitization as well as intracellular signaling in the nucleus accumbens, caudate/putamen and hippocampus (Zhu et al., 2012; Chen et al., 2018). Regarding the role of D_3_R in alcohol consumption, D_3_R KO mice show either little ethanol intake in both two-bottle choice and Drinking in the Dark (DID) paradigms (Leggio et al., 2014), or no differences in ethanol intake compared to wild type controls in the two-bottle drinking procedure, conditioned place preference, or in an operant self-administration paradigms (Boyce-Rustay and Risinger, 2003). In other studies, D_3_R KO showed lower ethanol intake and more severe ethanol withdrawal following 4 days of treatment with 7% ethanol in the liquid diet method (Narita et al., 2002), as well as attenuated behavioral sensitization to ethanol (Harrison and Nobrega, 2009). These findings suggest D_3_R modulates physical ethanol dependence and that D_3_R-KO mice may be more sensitive to alcohol (Narita et al., 2002). As such, the D_3_R may play different roles in ethanol intake vs. psychostimulant and opioid intake.

### D_4_ Receptors

The D_4_R is another inhibitory G_*i*_-coupled dopamine receptor expressed at relatively low levels in the cortex, amygdala, hippocampus, and the pituitary (Valerio et al., 1994; Asghari et al., 1995; Mrzljak et al., 1996; Primus et al., 1997; see Di Ciano et al., 2014 for review). Alleles with variable numbers of tandem repeats (VNTR) have been detected in the *DRD4* gene and may be linked to traits associated with drug use, including impulsivity, novelty seeking, and risk taking (Benjamin et al., 1996; Ptacek et al., 2011; Di Ciano et al., 2014). There is an inconsistent literature on *DRD4* VNTR and SUD, with some studies reporting *DRD4* allele variations in people with alcohol, nicotine, methamphetamine or opioid use disorders, while other studies reporting weak or no associations (see McGeary, 2009; Chen et al., 2011; Di Ciano et al., 2014).

Deletion of the D_4_R in mice increased locomotor sensitivity to ethanol, cocaine, and methamphetamine, and elevated dopamine synthesis and turnover in the dorsal striatum (although some studies also report reduced dopamine levels in the striatum/nucleus accumbens following D_4_R deletion; Rubinstein et al., 1997; Katz et al., 2003; Thomas et al., 2007). Cocaine produces stronger discriminative-stimulus like effects in D_4_R KO mice compared to controls (Katz et al., 2003). However, in i.v. self-administration studies, D_4_R KO mice showed no differences in responding for cocaine (or food) compared to wild type controls (Thanos et al., 2010b). D_4_R KO mice do show enhanced methylphenidate and amphetamine place preferences compared to wild type controls, but there were no genotypic differences in cocaine place preferences (Thanos et al., 2010a). Few differences have been reported between D_4_R KO mice and wild type mice in ethanol consumption and ethanol preferences in a two-bottle choice test (Falzone et al., 2002). These findings suggest that reduced D_4_R expression is unlikely to be a risk factor for the development of SUD.

### D_5_ Receptors

To our knowledge, very little is known about the functional role of the D_5_R in AUD and SUD. The D_5_R is expressed at low levels in the prefrontal cortex, substantia nigra, hypothalamus, and hippocampus, as well as the caudate and nucleus accumbens (Ciliax et al., 2000; Beaulieu and Gainetdinov, 2011). D_5_R KO mice show stronger cocaine-induced locomotor activity compared to wild type controls, but no differences in cocaine discriminative-stimulus effects (Elliot et al., 2003) and normal cocaine place preferences and locomotor sensitization to cocaine (Karlsson et al., 2008). These findings suggest the D_5_R may play a limited role in cocaine-related behaviors (Karlsson et al., 2008).

## Decreased mGluR_2_ Expression Is a Risk Factor for Cocaine, Opioid and Alcohol Use

In addition to dopamine, the glutamate system plays a fundamental role in substance misuse and addiction (Kalivas, 2009). Glutamate receptors are classified into ionotropic and metabotropic. There are three groups of metabotropic glutamate receptors: Group 1 includes mGluR_1_ and mGluR_5_, Group 2 mGluR_2_, and mGluR_3_, and Group 3 mGluR_4_,_6_,_7_,_8_ (Caprioli et al., 2018). Among these glutamate receptors, mGluR_2_ is the most-well studied in AUD and SUD.

mGluR_2_ is one of the major presynaptic autoreceptors that modulates glutamate release (Baker et al., 2002; Xi et al., 2002; Moussawi and Kalivas, 2010; Caprioli et al., 2018). While data in humans are lacking, rats with a history of cocaine self-administration or cocaine exposure show increases in mGluR_2/3_ density in the prefrontal cortex and nucleus accumbens during exposure, but decreases in mGluR_2/3_ expression in these same brain regions during extinction or withdrawal (Ghasemzadeh et al., 2009; Pomierny-Chamiolo et al., 2017; also see Xi et al., 2002). mGluR_2/3_ agonists and positive allosteric modulators reduce dopamine and glutamate release and inhibit cocaine, nicotine and alcohol self-administration (Greenslade and Mitchell, 2004; Moussawi and Kalivas, 2010; Zhou et al., 2013; Johnson and Lovinger, 2015).

Both mouse and rat mGluR_2_ knockouts have been developed. Mice with deletion of mGluR_2_ show stronger cocaine conditioned place preferences and cocaine-induced locomotor sensitization than wild type mice (Morishima et al., 2005). mGluR_2_ KO mice also showed increased extracellular dopamine and glutamate release in the nucleus accumbens following cocaine administration compared to wild type controls (Morishima et al., 2005). Rats with deletion of mGluR_2_ show faster acquisition of cocaine self-administration and increased intake across multiple doses of cocaine (Yang et al., 2017). However, mGluR_2_ KO rats exhibit reduced responding when response requirements are higher and attenuated cocaine seeking during extinction and cocaine-primed reinstatement testing (Yang et al., 2017). mGluR_2_ KO rats also exhibit enhanced cocaine-induced dopamine increases in the nucleus accumbens, but suppressed cocaine-induced glutamate changes in this region. The behavioral effects of mGluR_2_ deletion may relate to a reduction in sensitivity to cocaine. Low mGluR_2_ expression could thus be a risk factor for cocaine addiction, particularly in the early stages or initial development of cocaine misuse (Yang et al., 2017).

With respect to opioids, mGluR_2_-deleted rats self-administer more heroin than wild type controls. As with cocaine, mGluR_2_ deletion is linked to exaggerated heroin-induced locomotor responses and higher dopamine and glutamate responses in the nucleus accumbens to heroin. However, mGluR_2_-deleted rats respond less for heroin than wild type controls under a progressive ratio schedule of reinforcement, and exhibited attenuated heroin seeking during extinction and heroin-primed reinstatement (Gao et al., 2018). Interestingly, mGluR_2_ KO rats express enhanced analgesic responses to morphine on the hot plate test, and more severe naloxone-precipitated morphine withdrawal signs. Together, these results suggest that mGluR_2_ mediates not only the rewarding aspects of heroin, but also analgesic and withdrawal-related effects (Gao et al., 2018), similar to findings with the D_3_R. mGluR_2_ deletion may enhance dopamine responses to misused drugs by causing a disinhibition of glutamate, and subsequently dopamine, release in the ventral tegmental area and other brain areas (Gao et al., 2018). As such, low mGluR_2_ expression may represent a risk factor for opioid as well as stimulant misuse.

Considering alcohol, genomic sequencing has revealed genetic variation in the mGluR_2_ gene, *Grm2*, that alters alcohol preferences in mice and rats (Zhou et al., 2013; Wood et al., 2017). Genetic manipulation to terminate mGluR_2_ expression (through insertion of a stop codon in *Grm2*) led to increased alcohol consumption and preferences in rats and mice in a two-bottle choice procedure and well as increased risk-taking behavior (Zhou et al., 2013; Wood et al., 2017). These observations indicate mGluR_2_ mediates alcohol reward in much the same way stimulant and opioid reward are mediated, such that low mGluR_2_ expression may constitute increased risk for alcohol misuse.

## Decreased M_4_R Expression Is a Risk Factor for Cocaine and Alcohol Use

The M_4_ muscarinic acetylcholine receptor is one of five muscarinic acetylcholine receptor subtypes. While the role of M_1_–M_3_ receptors in self-administration models has not been characterized, M_5_-KO mice displayed a decrease in cocaine self-administration (Fink-Jensen et al., 2003; Thomsen et al., 2005). As such, decreased M_5_ expression is unlikely to be a risk factor in the development of cocaine use disorder. Therefore, we focus on the M_4_ receptor here. M_4_-KO mice display an increase in cocaine self-administration compared to controls (Schmidt et al., 2011; Thomsen and Caine, 2016), similar to that observed in D_3_-KO mice described above. Increased cocaine self-administration in M_4_-KO mice was observed at intermediate doses of cocaine under both a FR and a PR schedule of reinforcement, manifested as an upward shift of cocaine self-administration dose-response curves (Schmidt et al., 2011). In agreement with these results, cocaine-induced increases in both nucleus accumbens dopamine levels and locomotor activity were also augmented in M_4_-KO mice compared to wild type mice (Schmidt et al., 2011). In addition, M_4_ KO mice consume more alcohol in a sucrose fading procedure and take longer to extinguish alcohol seeking (De La Cour et al., 2015). Taken together, these findings suggest that decreased M_4_R expression may promote cocaine or alcohol use.

In the brain, M_4_ is expressed in the striatum, cortex, hippocampus, and midbrain (Weiner et al., 1990; Sugaya et al., 1997; Schmidt et al., 2011), and in particular on D_1_R-expressing neurons in the striatum (Weiner et al., 1990). Accordingly, in M_4_ KO animals the lack of M_4_ receptors co-localized with D_1_ receptors on GABAergic projections may indirectly activate dopaminergic signaling (Tzavara et al., 2004), which may in part explain the enhanced cocaine effects observed in M_4_-KO mice.

## Reduced α_5_ Nicotinic Acetylcholine Receptor Expression is a Risk Factor For Cocaine and Alcohol Use

Nicotinic acetylcholine receptors (nAChRs) are pentameric ligand-gated ion channels composed of α (α_2_–α_7_, α_9_, and α_10_) and β (β_2_–β_4_) subunits that co-assemble in various combinations with distinct functional properties (Changeux, 2010). Several large-scale human GWAS identified variants in the α_3_, α_5_, and β_4_ nAChR subunits that increase the risk for nicotine dependence (Bierut et al., 2007, 2008; Sherva et al., 2008). One of these variants found in exon 5 of the α_5_ gene SNP (α_5_-SNP) has been recently introduced into the genome of the rat, resulting in increased nicotine self-administration at high doses and relapse behaviors (Forget et al., 2018). Consistent with these findings, α_5_ KO mice also show increased nicotine intake, an effect that is reversed by α_5_ re-expression in the medial habenula (Fowler et al., 2011).

In candidate gene association studies, α_5_-SNPs are associated with cocaine use disorder in humans with altered levels of α_5_ mRNA (Grucza et al., 2008; Wang et al., 2009; Sherva et al., 2010). Congruently, α_5_-KO rats showed a robust increase in cocaine-induced reinstatement of cocaine seeking, suggesting that reduced α_5_ expression may represent a novel biomarker for increased risk of relapse to cocaine use. Notably, the absence of the α_5_ nAChR subunit had no effect on the acquisition of cocaine self-administration or cocaine dose-response curves. Rats carrying the α_5_-SNPs showed impairment in the acquisition of cocaine self-administration (Forget et al., 2021). The precise mechanisms underlying α_5_ involvement in cocaine action remain unclear. Importantly, cocaine is also an inhibitor of nAChRs (Damaj et al., 1999; Francis et al., 2000). Thus, reduced α_5_ expression or α_5_-SNP in the mesolimbic dopamine system may differentially alter the dopamine response to cocaine and thus cocaine-seeking behaviors (Acevedo-Rodriguez et al., 2014).

With respect to alcohol, α_5_ KO mice show enhanced alcohol-induced hypothermia and anxiolysis, but reduced ethanol place preferences and reduced ethanol intake in the DID with restraint stress paradigm (Dawson et al., 2018). By contrast, α_5_ KO mice did not differ from wild type controls in the DID paradigm (Santos et al., 2013), although they were more sensitive to the acutely sedating effects of ethanol (Santos et al., 2013). Clearly, more research is needed to address the role of α_5_ nAchR in alcohol use disorder.

## Other Candidate Risk Genes for Drug Use

Other genes of interest implicated in substance misuse and addiction include the orphan G protein coupled receptor, GPR88, which is expressed in brain regions including the striatum, amygdala, and cortex (Mizushima et al., 2000; Becker et al., 2008; Ehrlich et al., 2018). GPR88 KO increases alcohol drinking and responding for alcohol (Ben Hamida et al., 2018), although to our knowledge little is known regarding the role of GPR88 in opioid or psychostimulant reward or seeking. Similarly, a point mutation in the μ opioid receptor gene, T394, alters μ opioid receptor internalization and tolerance to opioid analgesia. Induction of the T394 mutation in mice causes a loss of tolerance to the analgesic effects of opioids, increases i.v. heroin self-administration, and enhances heroin-induced dopamine release in the nucleus accumbens, but has little effect on cocaine self-administration (Wang et al., 2016). Few studies regarding T394 KO effects on other psychostimulant or alcohol reward have been conducted.

## Conclusions and Future Directions

Substantial evidence indicates a significant genetic component to the risk for addiction. In searching for genes that contribute to this risk, numerous approaches may be utilized to identify the genes underlying addiction susceptibility. In this article, several candidate genes have been evaluated and proposed as possible risk genes for the development of SUD. Identification of risk genes is based on a central assumption that if deletion or decreased expression of a gene leads to an increase in drug-taking and drug-seeking behaviors, such a gene variant may constitute a vulnerability for the early development of addiction. Table 1 summarizes the genes possibly contributing to the risk of SUD described above. However, we should point out that not all reports are consistent. This is a weakness in using a single gene-KO strategy to study complex behaviors that may result from gene–gene and gene–environment interactions. Thus, newer approaches to the study of addiction susceptibility should target multiple genes and multiple functional systems. In addition, our central hypothesis is based on findings in drug self-administration and reinstatement tests, which have been assumed to be the most reliable models to study drug addiction. However, other approaches to evaluate drug preference, tolerance vs. sensitization, and withdrawal syndromes may also be important in identifying addiction-related risk genes. Furthermore, it is not fully understood how such genetic variants (such as KO or decreased expression) convey susceptibility to addiction. We interpreted high rates of drug self-administration and increased intake as a compensatory response to attenuated reinforcing effects of drugs after deletion of one receptor gene, which, in concept, is similar to a well-accepted view that drug craving and compulsive drug seeking is closely associated with reward deficiency syndrome in drug users during abstinence, which has been considered to be a neurobiological trait for the diagnosis and treatment of impulsive, addictive, and compulsive behaviors (Blum et al., 2000, 2021; Gondré-Lewis et al., 2020).

**TABLE 1:** Summary of major findings from several gene knockout (KO) animals with increases in vulnerability to drug use.

Dopamine D_2_ receptor (D_2_R) KO mice
•↑ Cocaine self-administration (Caine et al., 2002) •↑ Cocaine conditioned place preferences (Bello et al., 2011). •↑ Dopamine response to cocaine (Rouge-Pont et al., 2002; Bello et al., 2011). •↑ Dopamine response to morphine (Rouge-Pont et al., 2002). •↓ Cocaine sensitization (Solis et al., 2021). •↓ Methamphetamine sensitization (Solis et al., 2021). • No change in morphine self-administration (Elmer et al., 2002). •↓ Food self-administration (Caine et al., 2002). •↓ Alcohol consumption (Phillips et al., 1998; Cunningham et al., 2000; Risinger et al., 2000). •↓ Feeding (Caine et al., 2002). •↓ Electrical Intracranial Self-Stimulation (ICSS; Elmer et al., 2005).
**Dopamine D_3_ receptor (D_3_R) KO mice**
•↑ Basal extracellular dopamine and locomotion (Koeltzow et al., 1998; Song et al., 2012). •↑ Locomotor response to novel environment (Accili et al., 1996; Boyce-Rustay and Risinger, 2003; Li et al., 2010). •↑ Cocaine self-administration (Song et al., 2012; but see Caine et al., 2012). •↑ Cocaine conditioned place preferences (Xu et al., 1997; Kong et al., 2011). •↑ Motivation for cocaine seeking, delayed extinction of cocaine conditioned place preferences (Xu et al., 1997; Kong et al., 2011). •↑ Cocaine hyperactivity (Xu et al., 1997). •↑ Cocaine sensitization (Carta et al., 2000; Chen et al., 2018). •↑ Methamphetamine sensitization (Carta et al., 2000; Chen et al., 2018; but see Harrison and Nobrega, 2009; Zhu et al., 2012). •↑ Cue-conditioned hyperactivity (Le Foll et al., 2002). •↑ Impulsive behavior in 5-choice serial reaction time task (Wang et al., 2017). •↑ Heroin self-administration (Zhan et al., 2018). • Mixed effects in alcohol preference, consumption or self-administration (Narita et al., 2002; Boyce-Rustay and Risinger, 2003; Leggio et al., 2014). •↑ Morphine analgesia (Li et al., 2012). •↓ Tolerance in morphine analgesia and withdrawal response (Li et al., 2012). •↑ Morphine conditioned place preferences (Narita et al., 2003; Frances et al., 2004). •↓ Methamphetamine hyperactivity (Carta et al., 2000; Chen et al., 2018). •↓ Morphine hyperactivity and sensitization (Li et al., 2010; Lv et al., 2019). •↓ Dopamine response to cocaine (Song et al., 2012). •↓ Dopamine response to heroin (Zhan et al., 2018).
**Dopamine D_4_ receptor (D_4_R) KO mice**
• No effect on cocaine self-administration (Thanos et al., 2010b). •↑ Cocaine hyperactivity (Rubinstein et al., 1997; Katz et al., 2003; Thomas et al., 2007). •↑ Methamphetamine hyperactivity (Rubinstein et al., 1997; Katz et al., 2003; Thomas et al., 2007). •↑ Cocaine discrimination (Katz et al., 2003). •↑ Methylphenidate conditioned place preferences (Thanos et al., 2010a). •↑ Amphetamine conditioned place preferences (Thanos et al., 2010a). •↑ Alcohol hyperactivity (Rubinstein et al., 1997; Katz et al., 2003; Thomas et al., 2007). • No effect on alcohol consumption (Falzone et al., 2002).
**Metabotropic glutamate receptor type 2 (mGluR_2_) KO rats or mice**
•↑ Cocaine self-administration (Yang et al., 2017). •↑ Cocaine conditioned place preferences (Morishima et al., 2005). •↑ Cocaine sensitization (Morishima et al., 2005). •↓ Cocaine seeking during extinction (Yang et al., 2017). •↓ Cocaine-induced reinstatement of drug seeking (Yang et al., 2017). •↑ Heroin self-administration (Gao et al., 2018). •↓ Heroin-induced reinstatement of drug seeking (Gao et al., 2018). •↓ Electrical Intracranial Self-Stimulation (ICSS; Yang et al., 2017). •↑ Opioid analgesia (Gao et al., 2018). •↑ Naloxone-precipitated withdrawal responses (Gao et al., 2018). •↑ Alcohol drinking and preference (Zhou et al., 2013; Wood et al., 2017). •↑ Risk taking and emotional behavior (Wood et al., 2017). •↑ Morphine hyperactivity and locomotor sensitization (Gao et al., 2018). •↑ Dopamine response to cocaine (Morishima et al., 2005; Yang et al., 2017). •↑ Glutamate response to cocaine (Morishima et al., 2005; but see Yang et al., 2017). •↑ Dopamine response to heroin (Gao et al., 2018). •↑ Glutamate response to heroin (Gao et al., 2018).
Muscarinic M_4_ acetylcholine receptor KO mice
•↑ Cocaine self-administration (Schmidt et al., 2011; Thomsen and Caine, 2016). •↑ Dopamine response to cocaine (Schmidt et al., 2011; Thomsen and Caine, 2016). •↑ Locomotor response to cocaine (Schmidt et al., 2011; Thomsen and Caine, 2016). •↑ Alcohol consumption (De La Cour et al., 2015).
α_5_ nicotinic acetylcholine receptor KO mice
•↑ Nicotine self-administration (Fowler et al., 2011). •↑ Nicotine-induced reinstatement of drug-seeking (Forget et al., 2018). • No change in cocaine self-administration (Forget et al., 2021). •↑ Cocaine-induced reinstatement of drug seeking (Forget et al., 2021). •↑ Alcohol-induced hypothermia and anxiolysis (Dawson et al., 2018). •↓ Alcohol consumption and preference (Dawson et al., 2018, but see Santos et al., 2013).
μ opioid receptor-T394A mutation
•↑ Heroin self-administration (Wang et al., 2016). • No effect on cocaine self-administration (Wang et al., 2016). •↑ Dopamine response to heroin (Wang et al., 2016). •↑ Locomotor response to heroin (Wang et al., 2016). •↓ Tolerance in opioid analgesia (Wang et al., 2016).

We should also point out other notable limitations in gene KO studies. For example, the majority of KO models described in this manuscript and elsewhere have lifelong deletion or interruption of the target protein, and compensatory up- or down-regulation of other genes, proteins and circuits in response to that lifelong deletion may occur that also impact behavior and brain function (El-Brolosy and Stainier, 2017). Furthermore, mouse genetic background can play an important role in the expression of behavioral phenotypes (Nelson and Young, 1998; Bothe et al., 2004, 2005; Babbs et al., 2018; Tuttle et al., 2018), and should be considered carefully when evaluating behavior in KO models. Behavioral phenotypes arise from interactions of hundreds of genes and thousands of gene variants functioning in large networks, rather than alterations in single genes giving rise to suppressed or elevated protein expression. As such, ongoing and future research efforts to uncover the genes and allelic variations that contribute to drug vulnerability include examination of highly diverse mouse strains, such as Collaborative Cross, Diversity Outbred, and Heterogenous Stock Collaborative Cross mouse populations (Bagley et al., 2021). Assessment of these genetically diverse populations reveals a range of drug-vulnerable and drug-resilient phenotypes, and subsequent genetic mapping efforts have already begun to identify novel genes and gene networks that mediate drug reward and drug seeking-related behaviors (Dickson et al., 2015; Schoenrock et al., 2020; Bagley et al., 2021). Conversely, reduced complexity crosses of behaviorally different, but genetically similar inbred rodent strains offer powerful means by which to identify new candidate genes and genetic loci contributing to drug reward, addiction and other complex traits (Bryant et al., 2020; Kantak et al., 2021).

While evidence is accumulating to support risk genes for addiction, at this time assays are unfortunately not yet widely available to clinicians or patients to identify whether a person exhibits low or high expression of a given risk gene informed by knockout mouse models. Moreover, extreme caution is warranted in using genetic information to inform mental health conditions and treatment decisions, and important ethical and moral implications must be considered in making gene-based assumptions (Chapman et al., 2018). However, recent efforts to develop Genetic Addiction Risk Scores may hold additional promise in helping people identify their genetic risk for addiction and to develop protective or resiliency factors to mitigate their vulnerability to substance misuse. We have added a reference that discusses Genetic Addiction Risk Scores as well as reward deficiency syndrome (Blum et al., 2014) as recommended. Identification of new candidate genes and proteins of interest will be critical to identify new medication development targets for the treatment and prevention of drug use and addiction.

## Author Contributions

CJ wrote the first draft. Z-XX designed research and revised the manuscript. Both authors contributed to the article and approved the submitted version.

## Conflict of Interest

The authors declare that the research was conducted in the absence of any commercial or financial relationships that could be construed as a potential conflict of interest.

## Publisher’s Note

All claims expressed in this article are solely those of the authors and do not necessarily represent those of their affiliated organizations, or those of the publisher, the editors and the reviewers. Any product that may be evaluated in this article, or claim that may be made by its manufacturer, is not guaranteed or endorsed by the publisher.
